# Metagenomic characterization of gut microbiota in patients with Disorders of Gut–Brain Interaction

**DOI:** 10.3389/fmicb.2026.1890519

**Published:** 2026-10-22

**Authors:** Wenhui Tan, Wenjing Shi, Jingwu Song, Xiaodong Chen, Meihong Lin, Yue Xu, Yikai Ling, Yao Ma, Yan Xia, Zhifeng Wang, Biao Liang, Xiaoqiao Yang

**Affiliations:** 1 Gastrointestinal Microecology Therapy Center, The Affiliated Guangdong Second Provincial General Hospital of Jinan University, Guangzhou, Guangdong, China; 2 01Life Institute, Shenzhen, China; 3 Shen 1001 Life Institute, Shanghai, China; 4 Research Institute of Tsinghua University in Shenzhen, Shenzhen, Guangdong, China; 5 Digestive Endoscopy Center, The Affiliated Guangdong Second Provincial General Hospital of Jinan University, Shenzhen, Guangdong, China

**Keywords:** Disorders of Gut–Brain Interaction, gut microbiota, irritable bowel syndrome, metagenomic sequencing, subtype

## Abstract

**Background:**

Patients with Disorders of Gut–Brain Interaction (DGBI) experience recurrent gastrointestinal symptoms without identifiable organic abnormalities. While earlier studies focused on neural and hormonal regulation, recent evidence suggests that gut microbiota dysbiosis and altered microbial metabolism may play key roles in DGBI.

**Methods:**

A total of 86 DGBI patients meeting the Rome V criteria were enrolled. Demographic data, disease duration, and symptom scores (Wexner Constipation Score, Self-Rating Anxiety Scale, and Abdominal Bloating Score) were collected. Fecal samples were subjected to metagenomic sequencing, followed by diversity, taxonomic, and functional analyses to characterize the gut microbiota profile of DGBI patients.

**Results:**

DGBI patients and healthy controls (HC) showed comparable α-diversity and no significant overall separation in β-diversity. At the genus level, Prevotella was more abundant in HC, whereas other dominant genera showed similar patterns between groups. Co-occurrence network analysis revealed altered microbial associations in DGBI. A species-level random forest model moderately discriminated DGBI from HC (AUC = 0.769). Among DGBI subtypes, FD showed lower species richness, while IBS-M exhibited higher microbial evenness and diversity. Subtype-specific microbial signatures were identified, including *Ruminococcus gnavus* in IBS-D, *Prevotella stercorea* and *Asaccharobacter celatus* in IBS-C, and *Victivallis vadensis* in FC. Moreover, exploratory analysis showed a negative correlation between *Asaccharobacter celatus* abundance and Wexner constipation scores in the IBS-C subtype.

**Conclusion:**

In conclusion, this study showed that patients with DGBI exhibited limited changes in overall gut microbial diversity compared with healthy individuals, but certain differences in microbial community patterns were observed across clinical subtypes, suggesting potential microbial ecological heterogeneity among different DGBI phenotypes.

## 1 Introduction

Formerly known as Functional Gastrointestinal Disorders (FGIDs), Disorders of Gut–Brain Interaction (DGBI) comprise a group of chronic gastrointestinal disorders characterized by recurrent gastrointestinal symptoms that arise from dysregulation of gut–brain interactions rather than structural abnormalities (Scarpellini et al., 2024). According to the Rome V criteria, DGBI are classified based on symptom patterns and include conditions such as irritable bowel syndrome (IBS), functional dyspepsia (FD), and functional constipation (FC). These disorders affect up to 40% of the global population and represent a significant burden on healthcare systems and patients’ quality of life (Sperber et al., 2021). Although DGBI are not life-threatening, their persistent symptoms such as abdominal pain, bloating, altered bowel habits, and early satiety can severely impair daily functioning and mental wellbeing (Fikree and Byrne, 2021).

Traditionally, research on DGBI has centered on neural and hormonal mechanisms. However, accumulating evidence highlights gut microbial dysbiosis—manifested as both structural imbalance and functional disruption—as a major contributor to the pathophysiology of DGBI (Wei et al., 2021). Transplantation of IBS patients’ fecal microbiota into germ-free mice reproduces altered gut motility and sensitivity, supporting the causative role of microbiota in gut-brain axis regulation (De Palma et al., 2017). Clinical studies consistently reveal DGBI patients exhibit reduced beneficial taxa (e.g., *Lactobacillus*, *Bifidobacterium*) and enrichment of potentially pathogenic bacteria (e.g., *Enterobacteriaceae*), highlighting microbiota as both a marker and mediator of disease state (Nafarin et al., 2019).

Emerging metagenomic and metabolomic approaches further reveal that microbial dysbiosis in DGBI is not limited to taxonomic shifts, but extends to profound alterations in microbial metabolic capacity, including short-chain fatty acid (SCFA) production, bile acid metabolism, and tryptophan catabolism (Xiao et al., 2021). Recent multi-omics studies integrating metagenomics, microbial transcriptomics, and metabolomics have demonstrated that DGBI-associated microbial alterations are characterized by coordinated changes in microbial composition, functional activity, and metabolic output. In patients with IBS, integrated multi-omics analyses identified subtype-specific microbial functional signatures, showing that IBS patients exhibited altered carbohydrate utilization, bile acid transformation, and amino acid metabolism pathways, which could distinguish different bowel habit phenotypes more effectively than taxonomic profiling alone (Jacobs et al., 2023). Similarly, combined transcriptomic and metabolomic profiling in post-infectious IBS revealed that microbial metabolic alterations were closely associated with host epithelial responses, including impaired mucosal barrier function and increased intestinal permeability, highlighting the importance of microbiota–metabolite–host interactions in disease pathogenesis (Caceres Lessa et al., 2025).

These microbial metabolites could exert direct effects on the enteric nervous system, immune cells, and smooth muscle, consequently modulating gastrointestinal motility, visceral sensitivity, and mucosal integrity (Soret et al., 2010). For instance, reduced SCFA levels in patients with IBS have been associated with impaired epithelial barrier function and aberrant motor activity (Waseem et al., 2023), while abnormal bile acid profiles can impact colonic transit and exacerbate symptoms of diarrhea or constipation (Fogelson et al., 2023). In functional constipation, recent integrated analyses combining microbiome, metabolome, and lipidome data further demonstrated that microbial dysbiosis is accompanied by alterations in energy metabolism, lipid metabolism, and bioactive metabolite profiles (Ryu et al., 2025). Collectively, these findings underscore microbial metabolism as a key mechanistic link between gut dysbiosis and functional gastrointestinal symptoms. Despite these insights, the precise structural and functional characteristics of gut microbial dysbiosis in patients with DGBI remain incompletely understood. It remains unclear whether specific microbial signatures or metabolic pathways are consistently associated with certain motility phenotypes, such as delayed gastric emptying or accelerated colonic transit.

Therefore, this study employs shotgun metagenomics to systematically characterize both gut microbial taxonomic and functional profiles in patients with DGBI. We aim to: (i) identify specific microbial species linked to abnormal motility patterns, taking advantage of metagenomic resolution beyond the limitations of 16S rRNA approaches (Jacobs et al., 2023). (ii) compare gut microbial profiles and their correlations with clinical symptoms across different DGBI subtypes, thereby providing insight into the potential microbial mechanisms underlying these disorders.

## 2 Methods

### 2.1 Patient participation

A total of 86 patients with Disorders of Gut–Brain Interaction (DGBIs) were recruited from Guangdong Second Provincial General Hospital according to the inclusion and exclusion criteria. All enrolled participants provided written informed consent, and the study was approved by the Ethics Committee of the Affiliated Guangdong Second Provincial General Hospital of Jinan University. Participants were required to discontinue any medications known to affect gastrointestinal function or gut microbiota composition, including but not limited to antibiotics, probiotics, and proton pump inhibitors. To comprehensively characterize the gut microbiota composition of these patients, we additionally obtained metagenomic sequencing data from another project registered in the National Center for Biotechnology Information database with the accession number PRJNA557323. After matching for age and body mass index, a total of 86 samples were selected as healthy controls (HC) for subsequent analyses. For each enrolled patient, demographic and anthropometric data—including age, sex, weight, and height—were recorded, and fecal samples were collected. During the outpatient visit, professional physicians evaluated each patient using the Wexner Scale, the Self-Rating Anxiety Scale (SAS), and the Abdominal Bloating Score. Collected fecal samples were used for metagenomic sequencing of gut microorganisms. Finally, the obtained metagenomic data were further analyzed through bioinformatics approaches, including diversity analysis, taxonomic annotation, and functional annotation.

Inclusion criteria: (I) Patients clinically diagnosed with DGBI according to the Rome V criteria (specific subtypes, such as IBS or functional constipation, should be recorded). (II) Age ≥18 years, with no restriction on gender, and capable of providing written informed consent. (III) Willingness to discontinue any medications that may affect gastrointestinal function or gut microbiota prior to study entry.

Exclusion criteria: (I) Presence of organic intestinal diseases or a history of recent major abdominal surgery. (II) Presence of severe cardiac, hepatic, neurologic, or endocrine diseases that, in the investigator’s judgment, may interfere with study assessments or pose safety risks. (III) Any other condition that, in the investigator’s opinion, makes the participant unsuitable for inclusion in this study.

### 2.2 Scale

#### 2.2.1 Wexner Scale

This scale includes eight items: bowel movement frequency, time per bowel movement, pain assessment, forms of assistance, completeness, number of unsuccessful attempts at bowel movement within 24 h, abdominal pain, and duration of condition. Each is scored according to severity or frequency, ranging from 0 (none) to 4 (very severe), with higher scores indicating more severe constipation. A total score of 15 or above is considered indicative of constipation, with higher scores reflecting a more severe condition (Agachan et al., 1996).

#### 2.2.2 Self-Rating Anxiety Scale

The Self-Rating Anxiety Scale (SAS) consists of 20 items, each rated on a 4-point Likert scale that primarily assesses the frequency of symptoms. The scoring criteria are as follows: a score of “1” indicates none or very little of the time; “2” indicates some of the time; “3” indicates most of the time; and “4” indicates almost all or all of the time. The main statistical indicator of the SAS is the total score. The raw score is obtained by summing the scores of all 20 items. The standard score is then calculated by multiplying the raw score by 1.25 and taking the integer part. Higher total scores indicate more severe anxiety. A standard score of 50 or above is considered indicative of anxiety, with higher scores reflecting greater severity (Zung, 1971).

#### 2.2.3 Abdominal Bloating Score

The Abdominal Bloating Score was developed for this study and consists of three dimensions: the frequency of abdominal bloating, the intensity of abdominal bloating, and the impact of abdominal bloating on daily life (Supplementary Table S1). The total score is obtained by summing the scores of the three dimensions, with a possible range of 1 to 13. Higher scores indicate more severe abdominal bloating. A score of 1 represents “no abdominal bloating and no impact,” while a score of 13 represents “persistent, extremely severe abdominal bloating with a severe impact on daily life.”

### 2.3 DNA extraction and metagenomic sequencing (DGBI samples)

Approximately 200 mg of stool was thoroughly homogenized with 0.5 M ethylenediaminetetraacetic acid (EDTA) at pH 8.0 and 550 μL of lysis buffer. Then, 20 μL of proteinase K and 20 μL of lysozyme were added to the mixture, which was incubated at 55 °C for 2 h. Following centrifugation at 13,400 g for 5 min, the supernatant was carefully collected, mixed with 5 M sodium chloride (NaCl), gently vortexed for 10 s, chilled at −20 °C for several minutes, and subsequently re-centrifuged at 13,400 g for 10 min. The supernatant was transferred to a clean Eppendorf (EP) tube, centrifuged at 13,400 g for 5 min, and then carefully aspirated into a new EP tube. Isopropanol was added, and the mixture was incubated at −20 °C for 20 min followed by centrifugation at 13,400 g for 10 min. Subsequently, the liquid was added without removing any precipitate. Thereafter, the precipitate was washed twice with 1 mL of 75% ethanol, and the residual liquid was removed by centrifugation followed by aspiration with a pipette tip. To prevent over-drying, the EP tube was left open under a clean bench for gentle air-drying. Finally, 50 μL of double-distilled water (ddH_2_O) was added to dissolve the DNA, followed by the addition of 1 μL of RNase A. The mixture was mixed and incubated at 37 °C for 15 min.

DNA concentration, purity, and integrity were evaluated using a Qubit 2.0 fluorometer and 1% agarose gel electrophoresis. Samples with a total DNA yield greater than 1 μg and an A260/A280 ratio between 1.8 and 2.0 were selected for sequencing. Library preparation was performed using the NEBNext® Ultra™ DNA Library Prep Kit, with unique index adapters assigned to each sample. Sequencing was carried out on an Illumina high-throughput platform. For library construction, genomic DNA was fragmented to approximately 350 bp via ultrasonication, followed by end repair, A-tailing, and adapter ligation via polymerase chain reaction (PCR). The amplified products were purified using the AMPure XP system. Library fragment size distribution was assessed with an Agilent 2,100 Bioanalyzer, and library concentration was quantified by qPCR to ensure sequencing quality. Sequencing was performed on an Illumina NextSeq platform.

### 2.4 Healthy control dataset and processing

HC data were obtained from a publicly available dataset. Genomic DNA was extracted using the Gentra Puregene Yeast/Bacteria Kit (QIAGEN, Germany). Library preparation and paired-end shotgun metagenomic sequencing were performed on an Illumina NovaSeq 6000 platform (Novogene HK). Subsequent data processing and bioinformatic analyses were conducted using the same pipeline as applied to the DGBI cohort.

### 2.5 Batch effect correction

Batch effects across studies were corrected using MMUPHin. Correction was performed separately for microbial taxonomic abundance profiles at the phylum, genus, and species levels and for functional profiles, including MetaCyc and KEGG Orthology (KO). Data source was specified as the batch variable, while the biological group of interest was retained as a covariate to preserve biologically meaningful differences. MMUPHin was used to estimate and remove systematic batch-associated shifts in feature abundance. The batch-corrected abundance matrices were subsequently used for statistical analyses and machine-learning modeling. Quality control (QC) of sequencing reads and samples.

### 2.6 Quality control (QC) of sequencing reads and samples

Adapter was trimmed and low-quality reads were filtered using trimmomatic (version 0.39). Then, host sequences were removed by aligning sequencing reads back to host genome reference (hg38) using soap2 (version2.20) when sequence identity exceeds 90% (Li et al., 2009). After quality control, the DGBI group achieved an average sequencing depth of 3.91 Gb per sample, while HC group achieved an average sequencing depth of 5.86 Gb per sample.

### 2.7 Taxonomic annotation of metagenome and abundance quantification

Taxonomic profiling of the metagenomic samples was performed using MetaPhlAn (version 3.0.9) (Blanco-Miguez et al., 2023) which uses clade-specific markers to provide pan-microbial (bacterial, archaeal, viral and eukaryotic) quantification at species-level. Functional pathway tables were generated with HUMAnN3 using the UniRef90 search mode and MetaCyc database to profile the functional potential of each metagenomic sample, followed by normalization via the relative abundance (relab) method (Beghini et al., 2021).

### 2.8 Statistical analysis

Alpha diversity, including observed species counts, Shannon, and Pielou indices, was quantified using an in-house Perl script, and differences were assessed with the Wilcoxon rank-sum test. Unless otherwise specified, all statistical analyses were conducted in R, and *p* values <0.05 were considered statistically significant. Beta diversity was evaluated by calculating Bray-Curtis distances with the Python module SciPy (version 1.5.1) (Virtanen et al., 2020) and visualized via principal coordinates analysis (PCoA) based on the resulting distance matrix using ggplot2 in R. PERMANOVA (permutation multivariate analysis of variance) was performed to evaluate the effects of different phenotypes on metagenomic profiles, using Bray-Curtis distances with 999 permutations implemented in the R package vegan.

Differentially enriched microbial taxa and functional pathways between DGBI and HC were identified using the Wilcoxon rank-sum test, restricted to those present in at least 10% of samples to reduce the impact of sparsely distributed observations. Multiple hypothesis testing was corrected using the Benjamini–Hochberg false discovery rate (FDR) procedure to control for type I errors.

Microbial co-occurrence networks were constructed based on Spearman’s rank correlations using the Hmisc package in R, and visualized in Cytoscape (v3.10.1) (Shannon et al., 2003). Networks were generated for species-level assignments of differentially abundant taxa across all samples as well as for functional pathways annotated by MetaCyc. In addition, group-specific networks were constructed for differentially abundant species within the DGBI cohort and their associations with clinical scores. To control for multiple testing, correlation *p* values were adjusted using the Benjamini–Hochberg procedure. Only associations with an absolute Spearman correlation coefficient ( ∣ρ∣ ) > 0.6 and a Benjamini–Hochberg-adjusted *p* value <0.05 were retained for network construction.

Random Forest (RF) classification was performed using the implementation available in the microeco (Liu et al., 2021) R package. At the species level, taxa were filtered to retain only those present in at least 70% of samples, thereby reducing the influence of sparsely distributed species. A simple random split was applied to the dataset, 70% of the samples were randomly selected for training, while the remaining 30% were reserved for independent testing and validation. Within the training dataset, Bayesian hyperparameter optimization combined with 5-fold cross-validation was performed to optimize the RF model parameters and improve model stability. Feature selection was subsequently conducted using the Boruta algorithm based on RF-derived feature importance to identify the most informative microbial biomarkers. The RF model was then reconstructed using the selected features and evaluated on the independent testing dataset. Model performance was assessed using receiver operating characteristic (ROC) curves.

Differentially abundant microbial taxa among different DGBI subtypes were identified using the LEfSe method implemented in the microeco R package. Taxonomic features were analyzed at the species level, and taxa with a linear discriminant analysis (LDA) score >3.0 were considered significantly discriminative. To control for false-positive findings caused by multiple comparisons, *p* values were adjusted using the Benjamini–Hochberg false discovery rate (FDR) correction, and only taxa with adjusted *p* values <0.05 were considered statistically significant. A total of 526 microbial features were included in the LEfSe analysis.

The relationships between LEfSe-identified differentially enriched taxa and clinical symptom scores were evaluated using Spearman correlation analysis. Correlation coefficients and corresponding *p* values were calculated, and significant associations were visualized using heatmaps. Associations between gut microbial community composition and clinical parameters were further assessed using distance-based redundancy analysis (dbRDA) based on Bray–Curtis dissimilarity. Clinical variables, including symptom severity scores and demographic characteristics, were incorporated as explanatory variables to evaluate their contribution to variations in microbial community structure.

## 3 Results

### 3.1 The baseline information and taxonomy characteristics of DGBI and HC

We recruited a total of 86 patients with DGBI, including 39 females. The baseline characteristics of the patients are presented in Table 1. The mean age (*p* = 0.7350) and mean BMI (*p* = 0.4544) of the DGBI and HC groups were similar, with no significant differences between them. The mean Wexner Scale score was 4.87 ± 6.34, the SAS score was 38.44 ± 5.11, and the Abdominal Bloating Score was 7.79 ± 3.23.

**Table 1:** General information of the patients.

Characteristics	DGBI	HC	*p* value
Number	86	86	\
Female, *n* (%)	39 (45.35%)	36 (41.86%)	\
Mean age, year (SD)	42.23 ± 13.68	43.64 ± 17.08	0.7350
Mean BMI (SD)	22.97 ± 3.18	22.86 ± 3.71	0.4544
Wexner scale (SD)	4.87 ± 6.34	\	\
Self-rating anxiety scale (SD)	38.44 ± 5.11	\	\
Abdominal bloating score (SD)	7.79 ± 3.23	\	\

The patients with DGBI were classified according to Rome V standard.

### 3.2 Differences in gut microbiota between DGBI and HC

To investigate the differences in gut microbiota between DGBI patients and HC, we performed metagenomic sequencing on fecal samples from both groups and analyzed the sequencing results. Species-level microbial profiling identified 376 taxa in the HC group and 474 taxa in the DGBI group, which were included in subsequent comparative analyses. The α-diversity analysis (Figure 1A) showed no significant difference in observed richness (*p* = 0.36), Pielou evenness (*p* = 0.24), or Shannon diversity (*p* = 0.55) between the DGBI and HC groups, indicating comparable species richness, evenness, and diversity between the two groups. Principal coordinate analysis (PCoA) based on Bray–Curtis distances (Figure 1B) showed substantial overlap between DGBI patients and HC (PERMANOVA, *R*^2^ = 0.0077, *p* = 0.165), indicating the broad similarity between the two groups. At the genus level (Figure 1C), the five most abundant genera in both groups were *Bacteroides*, *Prevotella*, *Eubacterium*, *Alistipes*, and *Faecalibacterium*. The relative abundance of *Prevotella* was higher in the HC group, whereas the other major genera showed broadly comparable abundance patterns between the DGBI and HC groups.

**Figure 1:**
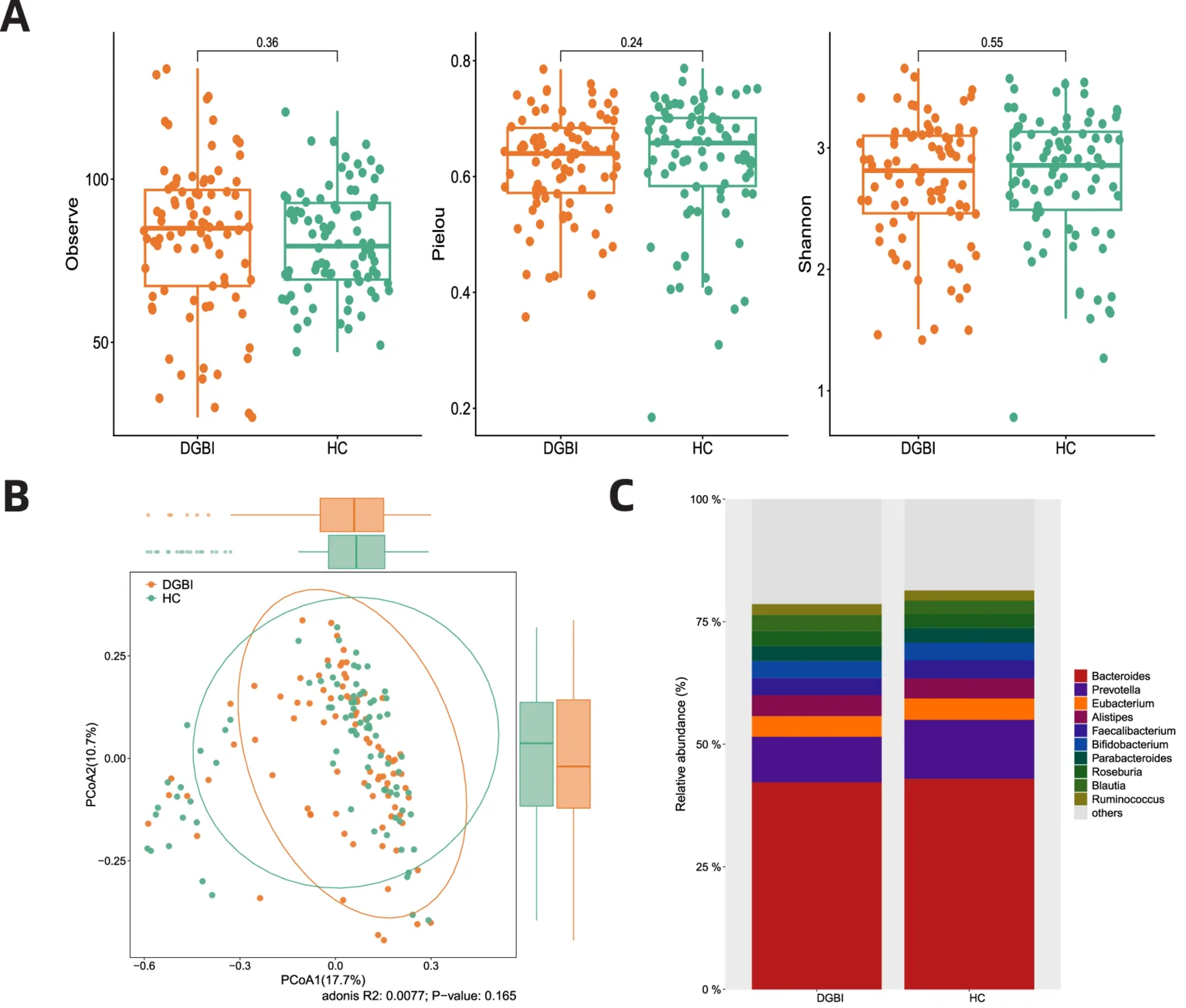
Comparison of gut microbial composition between DGBI patients and HC. **(A)** Comparison of Observed richness, Pielou evenness, and Shannon diversity indices. **(B)** Comparison of β-diversity between the two groups. **(C)** Bar chart showing the top 10 most abundant gut microbial genera in both groups.

Co-occurrence network analysis of interspecies correlations revealed distinct microbial association patterns between DGBI patients and healthy controls (Figure 2A). The networks included 52 nodes and 137 edges in network A and 24 nodes and 47 edges in network B. Compared with HC-associated taxa, DGBI-associated taxa, represented by *Clostridium citroniae* and *Hungatella hathewayi*, tended to cluster within a relatively compact microbial module, whereas HC-associated taxa, such as *Coprococcus comes* and *Oscillibacter* sp. 57_20, were located in a partially distinct module. These results suggest that DGBI may be associated with remodeling of interspecies ecological interactions rather than isolated changes in individual taxa. Species–pathway correlation analysis further showed that DGBI-associated microbial modules were connected with multiple metabolic pathways, whereas HC-associated taxa formed relatively smaller and more separated correlation modules (Figure 2B). Although co-occurrence network provide insights into potential ecological relationships, these analyses are based on statistical associations and cannot establish direct causal interactions.

**Figure 2:**
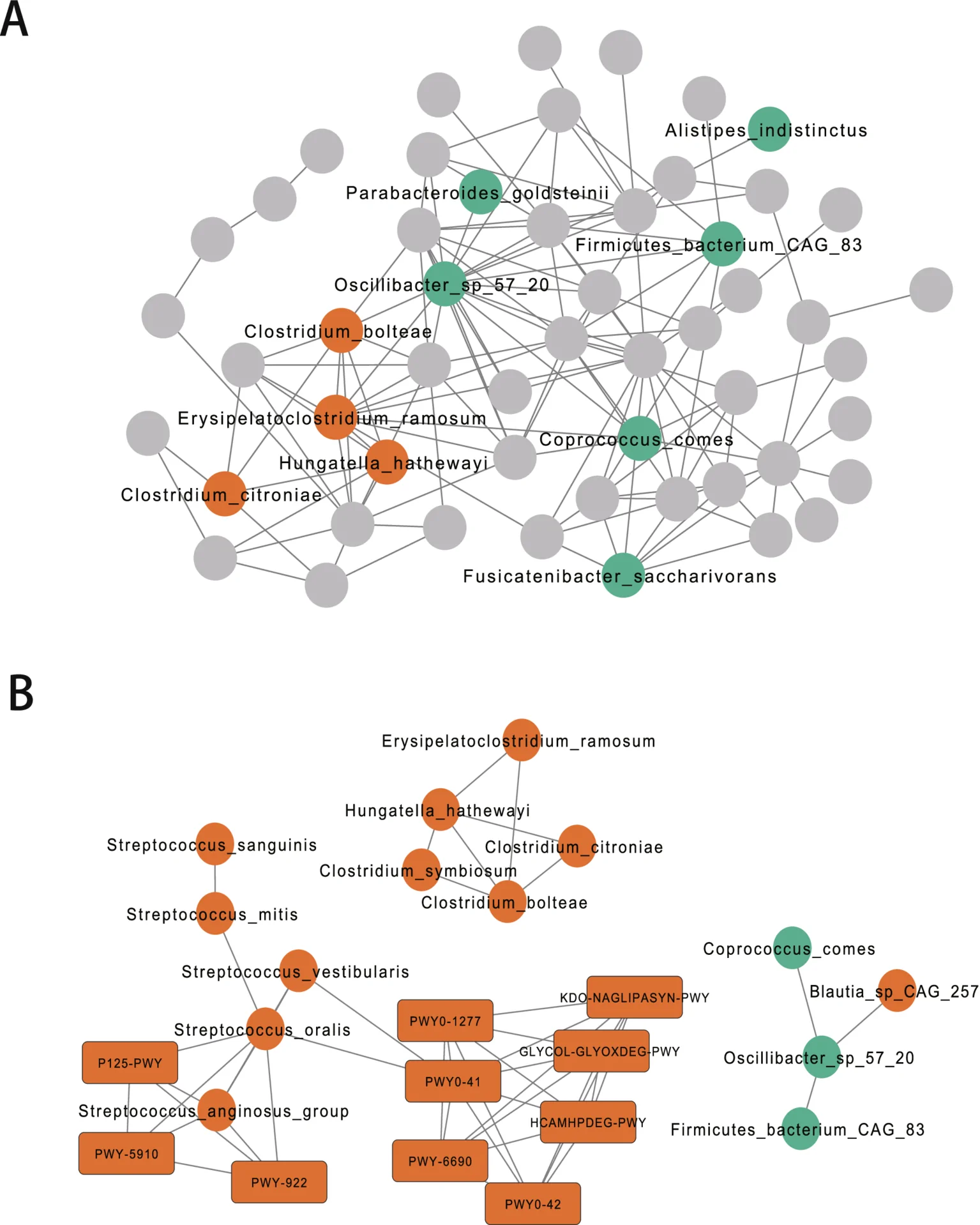
Co-occurrence network analysis reveals key bacterial taxa and functional pathways associated with the study phenotype. The network was constructed based on significant correlations (Spearman’s correlation coefficient |*ρ*| > 0.6, adjusted *p* < 0.05). The nodes and edges in the co-occurrence network represent microbial taxa, metabolic pathways, and their interactions, respectively. The node size represents the degree of connectivity, and node color indicates different microbial groups/pathway categories. **(A)** Co-occurrence network analysis of interspecies correlations. Colored nodes represent representative DGBI-associated or HC-associated taxa, and gray nodes represent other taxa included in the network. **(B)** Spearman correlation analysis between differential species and differential MetaCyc pathways across all samples. Circular nodes represent species, and square nodes represent MetaCyc pathways.

We constructed a random forest classifier to distinguish samples from the DGBI cohort and the external HC dataset and evaluated its performance in the test set. The confusion matrix showed that 18 DGBI samples and 20 HC samples were correctly classified, whereas 9 DGBI samples and 5 HC samples were misclassified (Figure 3A). The model achieved an overall accuracy of 73.1%, with a precision of 68.97%, recall of 80.00%, and F1-score of 74.07% when HC was treated as the positive class. ROC curve analysis further showed moderate discriminative performance, with an AUC of 0.769 (Figure 3B), indicating moderate classification performance between the two datasets. However, given the potential batch effects between the DGBI and HC groups, the current model should be regarded as exploratory, and its performance is insufficient to establish a clinically applicable diagnostic signature for DGBI.

**Figure 3:**
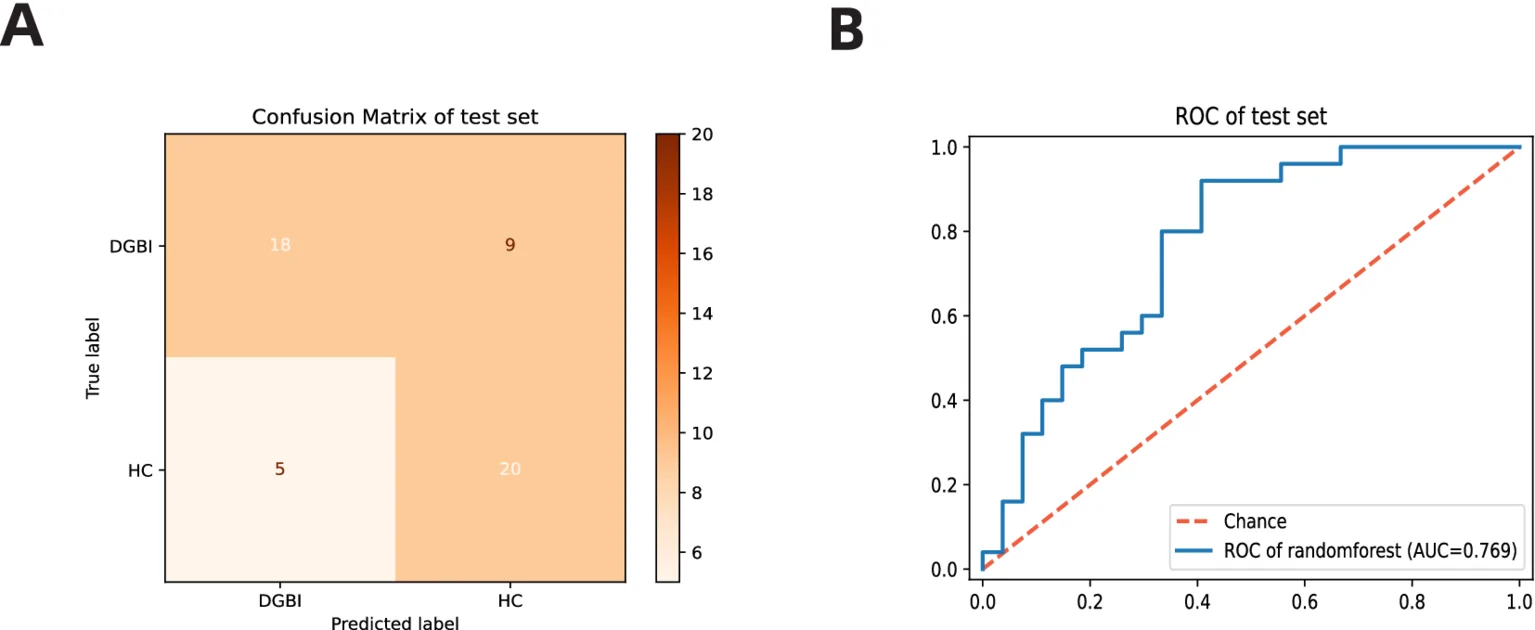
Construction and validation of the random forest model for DGBI patients and HC. At the species level, species with an occurrence rate of at least 70% were retained. Seventy percent of the samples were randomly selected for model training, and the remaining 30% were used for testing and validation. RF models were optimized by Bayesian hyperparameter tuning with 5-fold cross-validation. Key microbial features were identified using Boruta, and model performance was evaluated in an independent test set using ROC curves. **(A)** Confusion matrix of the random forest model. **(B)** ROC curve analysis of the random forest model.

Several MetaCyc metabolic pathways were differentially abundant between DGBI patients and healthy controls, as shown in Supplementary Figure S1. Functional annotation based on metagenomic sequencing data suggested that the DGBI group showed higher relative abundance of pathways related to bacterial cell envelope biosynthesis, carbon-source utilization, aromatic compound degradation, allantoin/urate-related metabolism, and isoprenoid precursor biosynthesis. Representative pathways included the lipid A/LPS-related biosynthesis pathway KDO-NAGLIPASYN-PWY, the peptidoglycan biosynthesis pathway PWY-5265, the glycol metabolism and degradation pathway GLYCOL-GLYOXDEG-PWY, and aromatic compound degradation pathways such as HCAMHPDEG-PWY, PWY-6690, and PWY0-1277. In contrast, the HC group showed higher relative abundance of ALLANTOINDEG-PWY, a pathway related to allantoin degradation. This analysis, based on metagenomic sequencing data and functional annotations from the MetaCyc database, provides an initial estimate of microbial functional potential, whereas actual metabolic activity remains to be further confirmed through metabolomic assessment.

### 3.3 Clinical indicators of different DGBI subtypes

Given the heterogeneity of DGBI symptoms and mechanisms, and the potential subtype-specific associations between gut microbiota and clinical manifestations, we grouped the DGBI data according to subtypes for analysis. There were 3 patients with IBS with predominant constipation (IBS-C), 14 with IBS with predominant diarrhea (IBS-D), 18 with IBS with mixed bowel habits (IBS-M), 5 with FC, 13 with FD, and 33 with functional bloating (FB). Exploratory analysis suggested that patients with diarrhea had significantly lower Wexner scores than those with constipation-type DGBI. Our finding shows that constipation patients have a Wexner score greater than 20, which is consistent with previous study (Wang et al., 2024). The Wexner scores of mixed-type IBS and functional bloating patients were similar and fell between those of the diarrhea and constipation groups. Anxiety and abdominal bloating scores were comparable among all DGBI subtypes, with no significant differences observed. In addition, no significant differences were observed among DGBI subtypes in BMI, age, SAS or bloating scores. In contrast, Wexner constipation scores differed significantly between subtypes: patients with FC and IBS-C scored notably higher than other subtypes, with scores exceeding 20, whereas differences among the remaining subtypes were modest, all below 10 (Supplementary Figure S2; Table 2).

**Table 2:** Clinical symptom scores of patients with different DGBI subtypes.

Subtype	Number	BMI	Age	Wexner score	SAS	Abdominal bloating score
IBS-C	3	23.59 ± 2.67	29.67 ± 4.03	22.7 ± 0.6	42.0 ± 6.1	8.3 ± 3.2
IBS-D	14	24.91 ± 3.51	37.86 ± 12.56	1.7 ± 2.3	37.2 ± 5.5	6.4 ± 4.5
IBS-M	18	22.39 ± 3.12	42.06 ± 14.56	4.6 ± 2.1	36.0 ± 3.3	6.3 ± 3.5
FC	5	22.31 ± 3.70	38.00 ± 15.76	23.4 ± 3.3	36.0 ± 2.6	9.8 ± 1.5
FD	13	24.00 ± 3.42	41.15 ± 14.55	1.2 ± 2.2	40.4 ± 4.7	8.9 ± 3.8
FB	33	22.62 ± 3.64	46.39 ± 11.56	3.4 ± 2.2	39.6 ± 5.6	8.4 ± 1.9

### 3.4 Microbial differences among DGBI subtypes

To explore the microbial differences among patients with different DGBI subtypes, metagenomic analysis of fecal samples was performed (Supplementary Figure S3). Species-level microbial profiles differed across DGBI subtypes, with 382, 199, 268, 168, 277, and 299 detected taxa in FB, FC, FD, IBS-C, IBS-D, and IBS-M groups, respectively. At the species level, α-diversity analysis revealed that patients with FD exhibited lower Observed richness, which was significantly reduced compared with that in IBS-C and IBS-M patients, whereas IBS-C patients showed relatively higher species richness. Meanwhile, IBS-M patients displayed significantly higher Pielou evenness and Shannon diversity indices than FB and FD patients. PCoA based on Bray–Curtis distances revealed no significant differences in β-diversity among the microbial communities at the phylum, genus, or species levels across the subtypes (PERMANOVA, *p* > 0.05).

The gut microbiota composition analysis at the species level (Figure 4A) revealed distinct microbial community profiles among different DGBI subtypes. *Bacteroides vulgatus* was the predominant species across all subtypes, showing relatively higher abundance in FC and FB patients. *Bacteroides uniformis* exhibited increased relative abundance in FC and FD patients, whereas *Prevotella copri* showed a higher proportion in FB patients but was not detected in IBS-C patients. *Bacteroides dorei* also showed a relatively lower abundance in FC patients. The beneficial bacterium *Faecalibacterium prausnitzii* displayed a higher relative abundance in IBS-C patients. To identify the microbial species contributing to inter-subtype differences, we performed LEfSe analysis, using effect size measurements to enrich bacterial taxa with different abundances among subtypes. At the species level (Figure 4B), LEfSe analysis identified distinct microbial signatures among different DGBI subtypes. The top five discriminative taxa with the highest LDA scores in IBS-C patients included *Prevotella stercorea*, *Ruminococcus torques*, *Bacteroides finegoldii*, *Asaccharobacter celatus*, and Alistipes timonensis. In addition, *Victivallis vadensis* showed strong discriminatory power and was primarily associated with the FC subtype (LDA > 3.0. FDR < 0.05).

**Figure 4:**
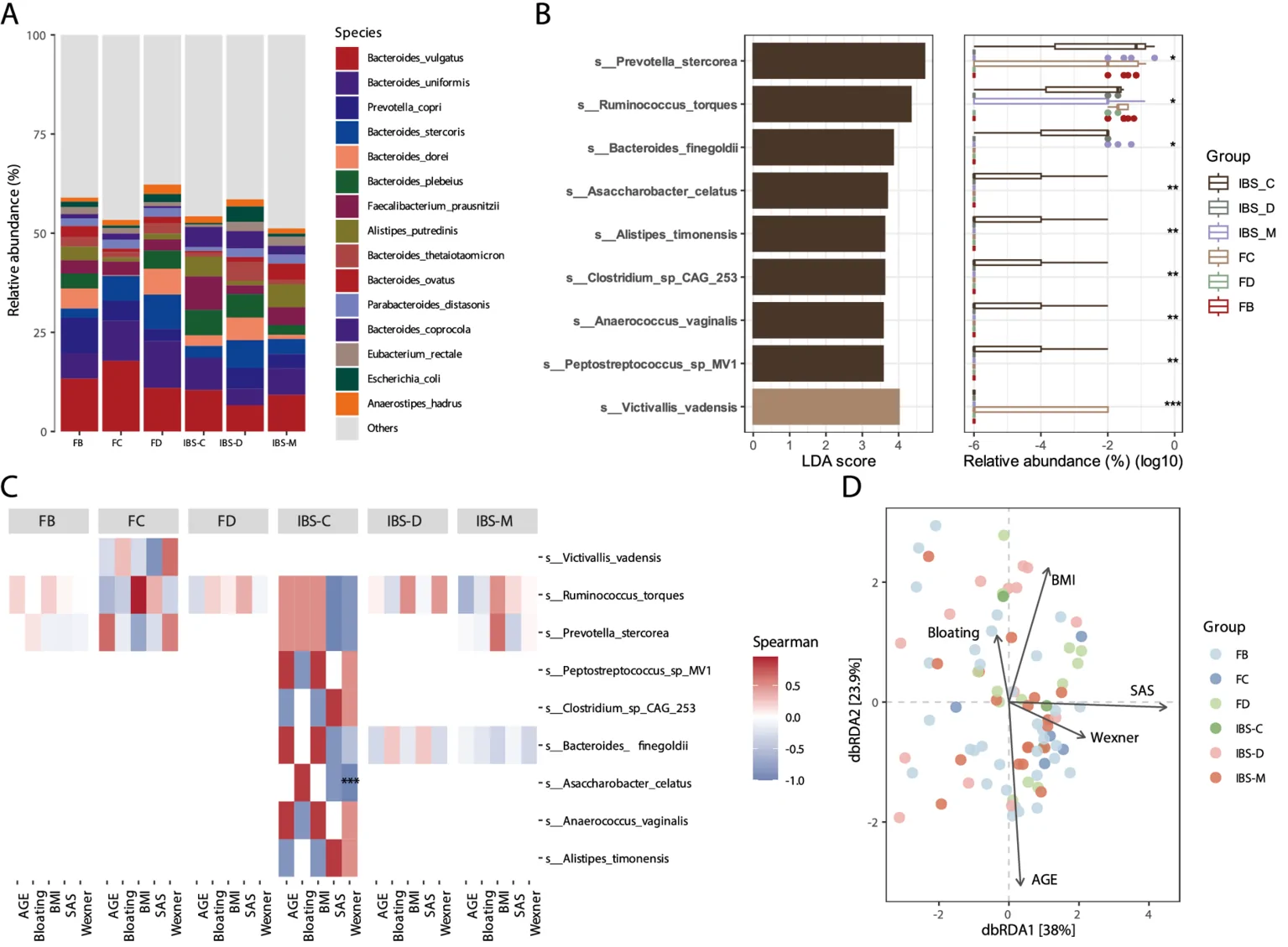
Microbial analysis of DGBI subtype. **(A)** Bar plot showing the top 15 abundant microbial species. **(B)** LEfSe analysis of gut microbiota among different DGBI subtypes using an LDA score threshold >3.0 to identify significantly differential taxa. **(C)** Heatmap showing correlations between clinical symptoms and LEfSe-identified taxa. **(D)** Distance-based redundancy analysis (dbRDA) showing associations between clinical variables and gut microbial community composition.

In different subtypes of DGBI patients, we further analyzed the correlations between gut microbiota at the genus level and symptom scores and performed dbRDA to assess the relationships between gut microbial community structure and clinical variables. In the exploratory correlation analysis, *Asaccharobacter celatus* in the IBS-C subtype showed a negative correlation with the Wexner constipation score (Figure 4C). Given the very small sample size of this subtype, this finding should be interpreted with caution. Furthermore, distance-based redundancy analysis (dbRDA) demonstrated that clinical parameters, particularly anxiety score, body mass index (BMI), and Wexner constipation score, were closely associated with variations in gut microbial community structures across different DGBI subtypes (Figure 4D). Given the relatively limited sample sizes of some DGBI subtypes, the microbiome comparisons among subtypes, including LEfSe and Spearman correlation analyses, were considered exploratory analyses aimed at identifying potential microbial patterns rather than establishing definitive subtype-specific signatures. These findings require further validation in larger independent cohorts.

## 4 Discussion

This study first compared the gut microbial structure between DGBI patients and HC, aiming to identify specific microbial species associated with abnormal gastrointestinal function. The comparison showed that there were no significant differences in α-diversity between DGBI patients and HC. However, this finding contrasts with several previous studies on IBS that have reported reduced microbial diversity. Nevertheless, the findings regarding alpha diversity in IBS remain inconsistent. A systematic review of gut microbiota studies in IBS patients indicated that alterations in alpha diversity varied considerably among studies, with some studies reporting reduced diversity, whereas others showing no significant differences compared with healthy controls (Goodoory et al., 2023). These inconsistencies may be related to differences in study populations, disease subtypes, methodological approaches, and particularly disease heterogeneity.

DGBI represents a highly heterogeneous group of Disorders of Gut–Brain Interaction, including IBS and other subtypes such as functional abdominal bloating, functional constipation, and functional diarrhea (Drossman and Hasler, 2016). Different DGBI subtypes may exhibit distinct patterns of microbial alterations; therefore, combining patients with different subtypes into a single cohort may attenuate the overall differences in microbial diversity. Previous studies have suggested that microbiota alterations associated with functional gastrointestinal disorders involve changes in specific taxa and functional pathways rather than a consistent reduction in overall microbial diversity (Simren et al., 2013). Thus, the absence of significant differences in α-diversity does not necessarily indicate the absence of gut microbiota alterations in patients with DGBI (Pittayanon et al., 2019).

β-diversity analysis showed no significant separation, though a numerically small effect size (*R*^2^ = 0.0077) was observed. Although these differences may reflect underlying biological variations, the potential influence of technical factors cannot be completely excluded, as the healthy control data in this study were obtained from a publicly available dataset. Differences in DNA extraction methods, library preparation procedures, and sequencing platforms across studies may introduce batch effects and potentially contribute to the observed microbial differences.

The comparison of specific gut microbial compositions between DGBI patients and HC was subsequently performed. At the genus level, the five most abundant genera in both groups were Bacteroides, Prevotella, Eubacterium, Alistipes, and Faecalibacterium. Most bacterial genera exhibited comparable relative abundances between the two groups; however, the relative abundance of Faecalibacterium was markedly lower in the DGBI group than in HC. Faecalibacterium is considered a beneficial commensal bacterium that contributes to the maintenance of intestinal microbial homeostasis. Previous studies have consistently reported a reduced abundance of Faecalibacterium in patients with IBS compared with healthy individuals. In a multi-omics study based on 16S rRNA sequencing, Chen et al. (2023) identified Faecalibacterium as one of the genera depleted in IBS patients among a cohort including 708 patients and 354 healthy controls. The same study further demonstrated that Faecalibacterium was among the top ten core taxa identified by co-occurrence network analysis across all IBS subtypes. Additionally, another study reported a negative correlation between Faecalibacterium abundance and IBS severity in humans (Rajilic-Stojanovic et al., 2011) suggesting that alterations in Faecalibacterium may contribute to the development and progression of IBS.

DGBI include multiple subtypes, which are likely related with differences in gut microbial composition (Black et al., 2020; Karakan et al., 2021; Carco et al., 2020). Therefore, we compared the gut microbial composition of different DGBI subtypes (IBS-D, IBS-C, IBS-M, FC, FD, FB) and analyzed the Spearman correlation between species-level gut microbiota and clinical indicators. Specifically, FD patients exhibited lower Observed species richness, whereas IBS-C patients showed higher species richness. Meanwhile, IBS-M patients displayed higher Pielou evenness and Shannon diversity indices. Previous studies have indicated that alterations in gut microbial diversity among DGBI patients do not follow a uniform pattern but may be influenced by multiple factors, including disease subtype, symptom phenotype, and intestinal transit characteristics. For example, IBS patients have generally been reported to exhibit reduced microbial richness and diversity; however, substantial differences exist among IBS subtypes. IBS-D patients may experience altered microbial colonization environments due to accelerated intestinal transit, resulting in reduced microbial richness and ecological stability, whereas constipation-predominant patients may develop distinct microbial ecosystems due to prolonged intestinal retention time (Pittayanon et al., 2019; Jeffery et al., 2020). Overall, our findings support the concept that DGBI is not characterized by a single pattern of microbial dysbiosis but rather encompasses diverse microbial ecological states driven by different clinical subtypes.

We further analyzed species-level gut microbial differences across DGBI subtypes and the Spearman correlation between species abundance and symptom scores. The results showed that *Prevotella copri* was significantly decreased in IBS-C patients, whereas *Bacteroides dorei* was also significantly reduced in FC patients, suggesting that different DGBI subtypes may exhibit distinct species-level microbial alterations. *Prevotella copri* is involved in dietary polysaccharide degradation and short-chain fatty acid metabolism, and changes in its abundance may influence the intestinal metabolic environment and functional status (Kovatcheva-Datchary et al., 2015). *Bacteroides dorei*, as a common intestinal commensal bacterium, participates in polysaccharide metabolism and host immune regulation, and its reduction may be associated with alterations in intestinal ecological stability (Wexler, 2007).

Exploratory Spearman correlation analysis showed a negative correlation between the abundance of *Asaccharobacter celatus* and the Wexner constipation score in IBS-C patients. *Asaccharobacter celatus* belongs to the family Coriobacteriaceae and is an obligate anaerobic bacterium capable of participating in intestinal equol biosynthesis. Previous studies have demonstrated that members of the Coriobacteriaceae family contribute to host–microbiota interactions by modulating metabolite production, immune responses, and intestinal ecological homeostasis (Liu et al., 2018). In the present study, this association may reflect a potential relationship between *Asaccharobacter celatus* and constipation-related clinical features. However, given the very small sample size of the IBS-C subtype and limited direct evidence regarding the role of *Asaccharobacter celatus* in functional gastrointestinal disorders, this finding should be interpreted cautiously and requires further validation in larger cohorts and functional studies.

Although this study revealed differences in gut microbiota composition between DGBI patients and healthy individuals, as well as distinct microbial characteristics among different subtypes, it still has some limitations. First, although MMUPHin correction can reduce systematic technical biases, residual confounding caused by differences in sample collection, DNA extraction, and sequencing platforms cannot be completely excluded. Therefore, the comparisons between DGBI patients and HC should be interpreted cautiously and require further validation in larger cohorts with standardized sample collection and sequencing protocols. Additionally, the relatively small sample sizes of some DGBI subtypes, particularly constipation-predominant subtypes, may limit the statistical power and robustness of subtype comparisons. Therefore, these findings should be interpreted as exploratory results. Further recruitment of constipation-predominant DGBI patients and validation in larger independent cohorts will be necessary to confirm these observations. Finally, the Abdominal Bloating Score was developed for this study and has not been formally validated; consequently, all findings based on this measure should be interpreted as exploratory.

## 5 Conclusion

In summary, DGBI patients showed no significant differences in overall microbial diversity or community structure compared with healthy individuals, but subtle alterations were observed in taxonomic composition, functional profiles, and alpha diversity across certain subtypes, suggesting potential microbial ecological heterogeneity among clinical phenotypes. These findings support specific microbiota-related alterations in DGBI rather than pronounced global dysbiosis and warrant further validation in larger cohorts.

## Data Availability

The datasets supporting the conclusions of this article are available in the European Nucleotide Archive (ENA) with the accession number PRJEB102751.
