# Supplementary material for: Metagenomic characterization of gut microbiota in patients with Disorders of Gut–Brain Interaction
Source: Front Microbiol. 2026 Oct 22;17:1890519. doi: 10.3389/fmicb.2026.1890519 (PMC13736836; doi:10.3389/fmicb.2026.1890519)
Supplement: Supplementary file 1 [file Supplementary_file_1.pdf]

Supplementary Information

Table S1. Abdominal Bloating Score

Assessment Dimension	Score Range	Grading Description and Corresponding Scores
Abdominal Bloating Frequency	1–5 points	1 point: Never experience abdominal bloating
		2 points: Occasional occurrence (less than once per month)
		3 points: Sometimes occurs (1–3 times per month, or less than once per week)
		4 points: Frequent occurrence (1–3 times per week)
		5 points: Always occurs (4 or more times per week, or persistent)
Abdominal Bloating Intensity	0–4 points	0 points: No sensation of abdominal bloating
		1 point: Mild bloating (noticeable but not uncomfortable)
		2 points: Moderate bloating (clearly perceived abdominal distension with mild discomfort)
		3 points: Severe bloating (obvious abdominal distension accompanied by marked discomfort)
		4 points: Extremely severe bloating (intense abdominal distension, severely affecting physical sensation and even intolerable)
Impact of Abdominal Bloating on Daily Life	0–4 points	0 points: No impact (normal work, eating, and activities are not affected)
		1 point: Mild impact (occasional need to adjust activities, such as reducing strenuous exercise)
		2 points: Moderate impact (some daily activities are limited, such as inability to stand for long periods or reduced food intake)
		3 points: Significant impact (most daily activities are disrupted, such as decreased work efficiency and reduced social activities)
		4 points: Severe impact (almost unable to carry out normal daily activities, requiring bed rest or frequent rest)

Total Score Calculation	1–13 points	The total score is obtained by summing the scores of the three dimensions. A score of 1 indicates “no abdominal bloating and no impact,” while a score of 13 indicates “persistent, extremely severe abdominal bloating with a severe impact on daily life.”

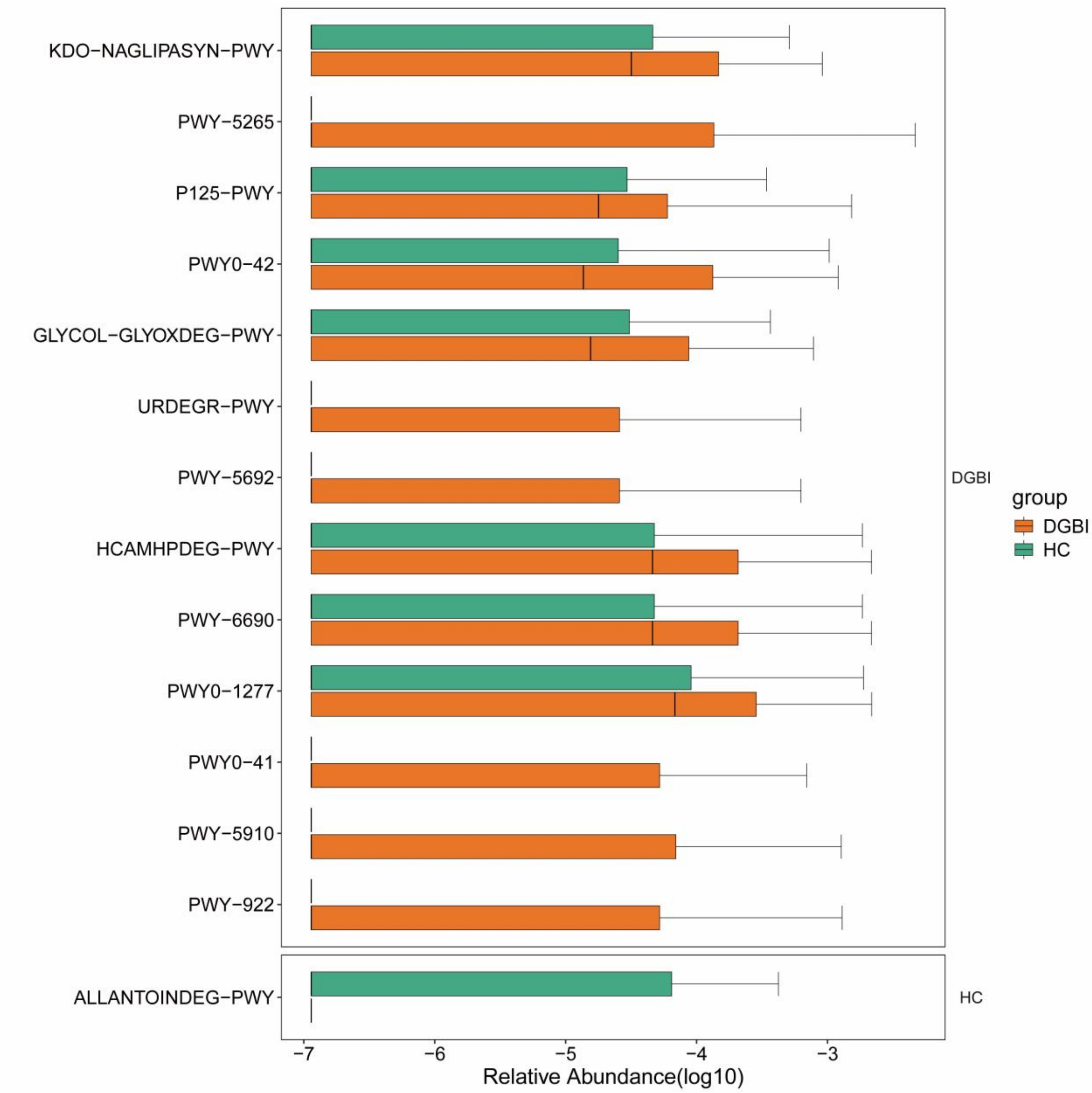

Figure S1. MetaCyc metabolic pathways showing differential abundance between DGBI patients and HC.

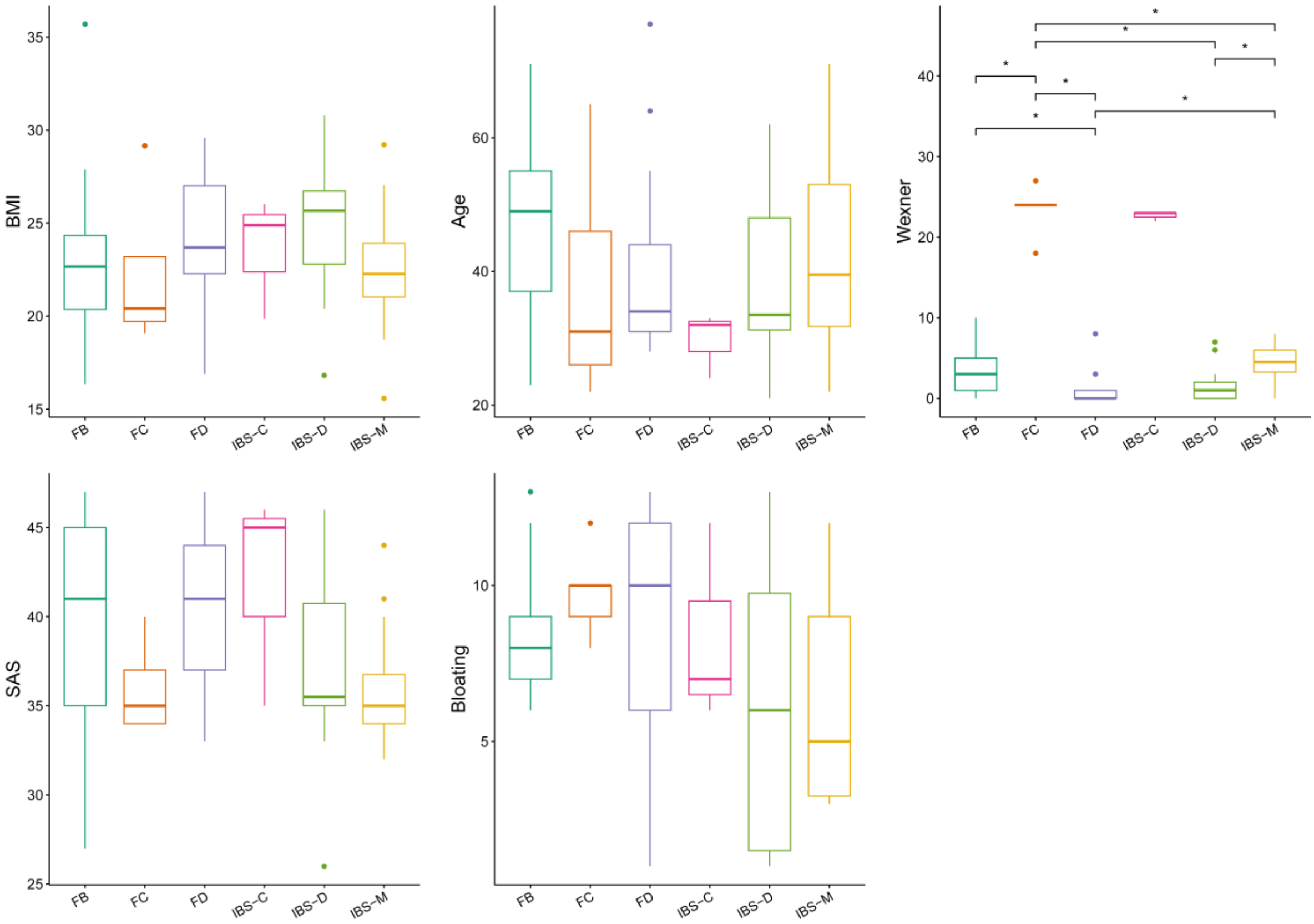

Figure S2. Statistical analysis of differences in various clinical indicators among different subtypes of DGBI.

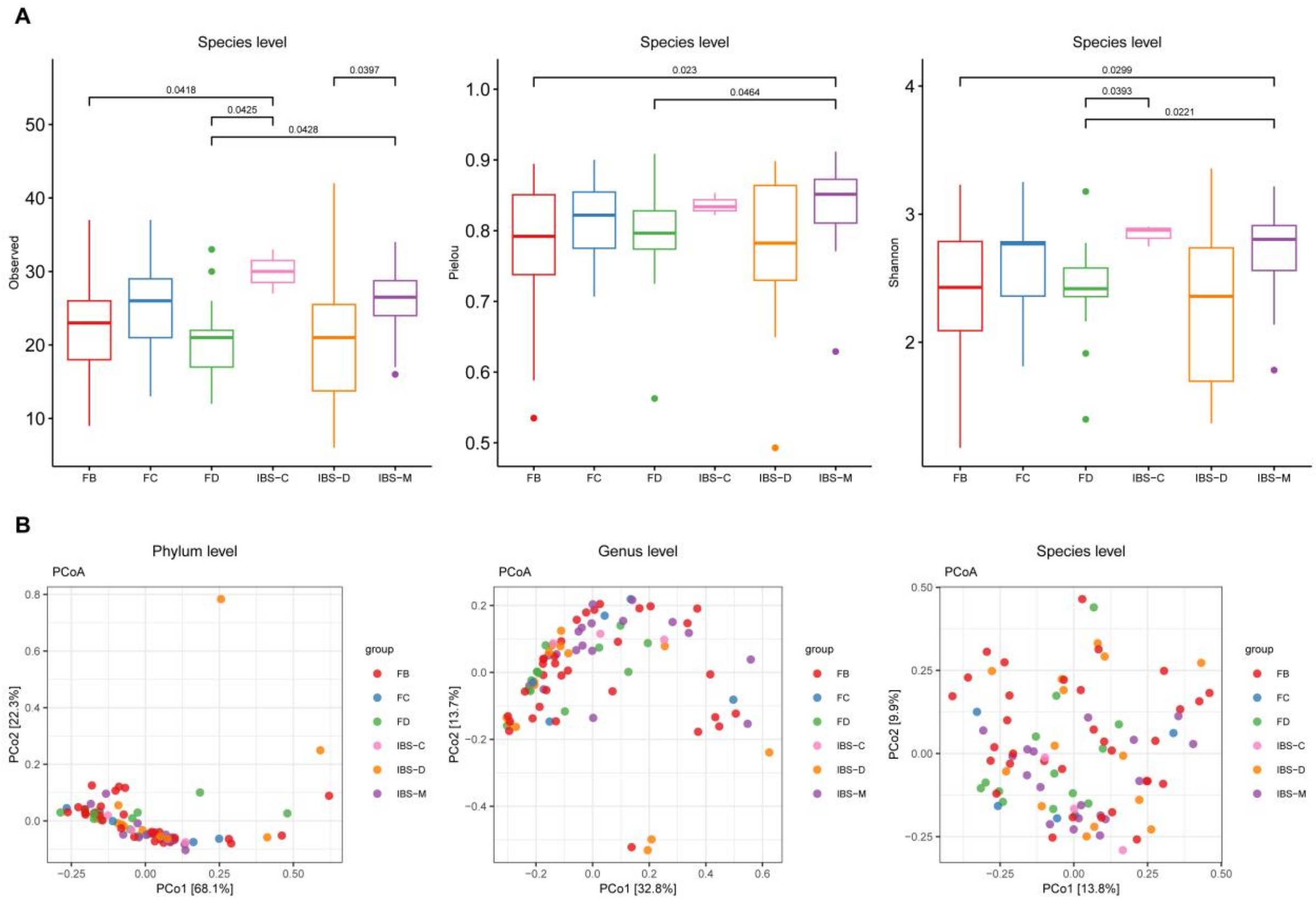

Figure S3. Comparison of gut microbial structure among different DGBI subtypes. (A) Comparison of Observed richness, Pielou evenness, and Shannon diversity indices. (B) β-diversity analysis of different DGBI subtypes at the phylum, genus, and species levels (From left to right are phylum, genus, and species, respectively).
